# Identification of *HOXD4* Mutations in Spinal Extradural Arachnoid Cyst

**DOI:** 10.1371/journal.pone.0142126

**Published:** 2015-11-06

**Authors:** Yoji Ogura, Noriko Miyake, Ikuyo Kou, Aritoshi Iida, Masahiro Nakajima, Kazuki Takeda, Shunsuke Fujibayashi, Masaaki Shiina, Eijiro Okada, Yoshiaki Toyama, Akio Iwanami, Ken Ishii, Kazuhiro Ogata, Hiroshi Asahara, Naomichi Matsumoto, Masaya Nakamura, Morio Matsumoto, Shiro Ikegawa

**Affiliations:** 1 Laboratory of Bone and Joint Diseases, RIKEN Center for Integrative Medical Sciences, Tokyo, 108–8639, Japan; 2 Department of Orthopaedic Surgery, School of Medicine, Keio University, Tokyo, 160–8582, Japan; 3 Department of Human Genetics, Yokohama City University Graduate School of Medicine, Yokohama, 236–0004, Japan; 4 Department of Orthopaedic Surgery, Kyoto University, Kyoto, 606–8507, Japan; 5 Department of Biochemistry, Yokohama City University Graduate School of Medicine, Yokohama, 236–0004, Japan; 6 Department of Orthopaedic Surgery, Saiseikai Central Hospital, Tokyo, 108–0073, Japan; 7 Department of Systems BioMedicine, Tokyo Medical and Dental University Graduate School of Medical and Dental Sciences, Tokyo, 113–8510, Japan; Innsbruck Medical University, AUSTRIA

## Abstract

Spinal extradural arachnoid cyst (SEDAC) is a cyst in the spinal canal that protrudes into the epidural space from a defect in the dura mater and leads to neurological disturbances. We previously showed that familial SEDAC is caused by *FOXC2* mutation; however, the causal gene of sporadic SEDAC has not been identified. To identify the causal gene of sporadic SEDAC, we performed whole exome sequencing for 12 subjects with sporadic SEDAC and identified heterozygous *HOXD4* loss-of-function mutations in three subjects. *HOXD4* haplo-insufficiency causes SEDAC and a transcriptional network containing *HOXD4* and *FOXC2* is involved in the development of the dura mater and the etiology of SEDAC.

## Introduction

**Fig 1:**
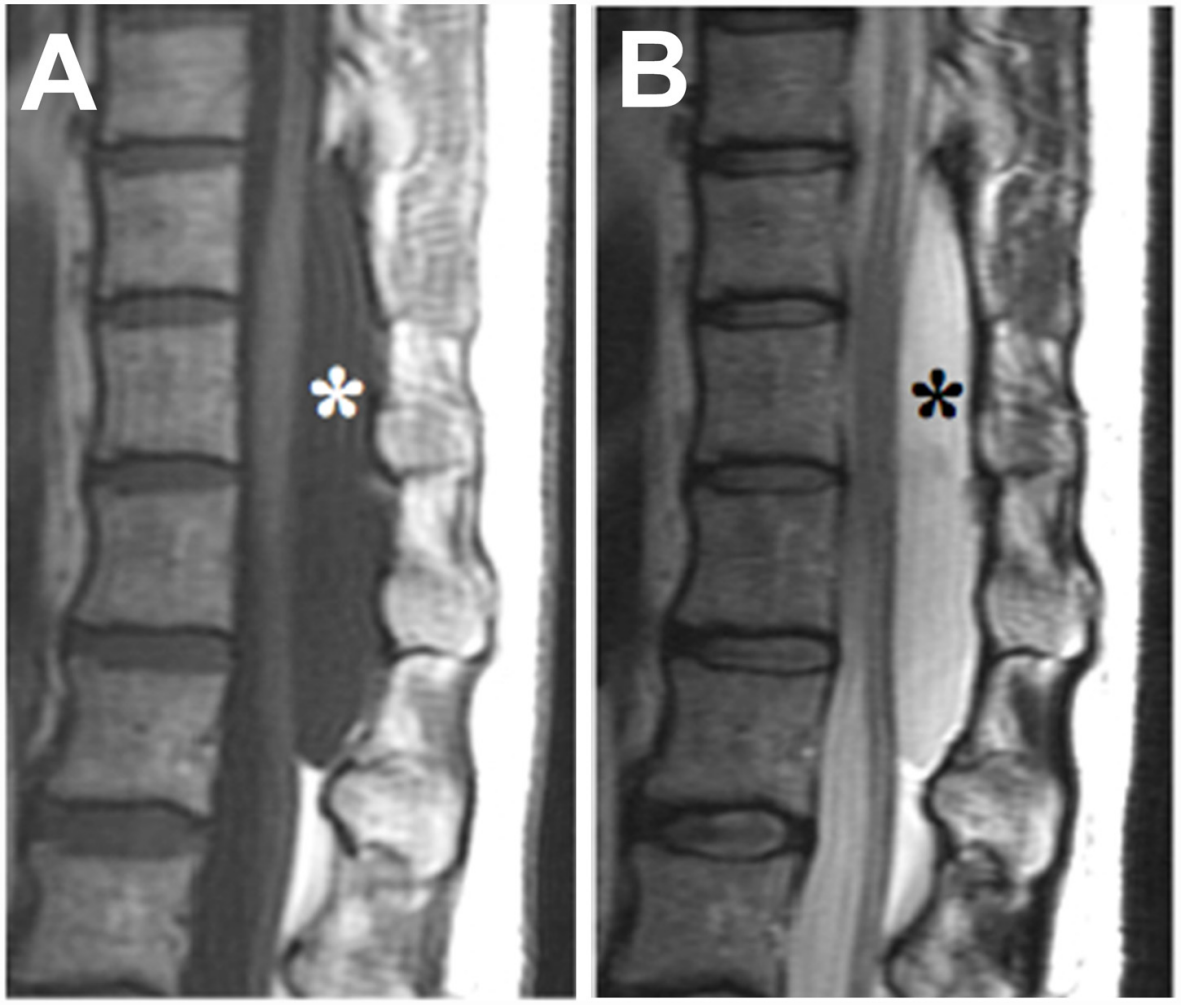
Spinal extradural arachnoid cyst. T1- (A) and T2- (B) weighted sagittal plane images of MRI scan. Subject P5. There is a single cyst (asterisk marks) dorsal to the spinal cord at the thoracolumbar spine.

Spinal extradural arachnoid cyst (SEDAC) is a cyst in the spinal canal (Fig 1), which is formed by arachnoid mater protruding from a dural defect that connects the intra- and epi-dural spaces. SEDAC represents 1% of all primary spinal tumours[1] and occurs predominantly in the lower thoracic to lumbar area, posterior to the spinal cord.[2] SEDAC expands due to retention of the cerebrospinal fluid in response to changes in spinal pressure. The enlarged cyst compresses the spinal cord and leads to neurological disturbances.[3] The onset is usually after middle age since the expansion progresses gradually.

SEDAC could be secondary to inflammation, previous spinal surgery and closed spinal trauma;[4] however, in most cases, it is caused by an idiopathic, congenital dural defect. Genetic etiology of SEDAC has been suggested.[5, 6] We previously investigated 2 familial SEDAC pedigrees associated with lymphedema-distichiasis syndrome (LDS) (OMIM 153400) and identified heterozygous loss of function mutations in *FOXC2* (forkhead box C2).[6] In the study, we also investigated seven sporadic SEDAC patients, but found no *FOXC2* mutation. Furthermore, clinical features of the sporadic SEDAC without a *FOXC2* mutation were significantly different from syndromic SEDAC with a *FOXC2* mutation in age of onset and number and location of the cysts,[6] suggesting genetic heterogeneity of SEDAC.

To identify the causal gene of sporadic SEDAC, we performed whole exome sequencing (WES) for 12 subjects with sporadic SEDAC. We identified *HOXD4* loss-of-function mutations in three subjects.

## Materials and Methods

### Subjects

We recruited 12 sporadic SEDAC subjects (Table 1). SEDAC was diagnosed using MRI. All subjects were Japanese and had no mutations or structural abnormalities in *FOXC2*. All but one received surgery. Secondary SEDAC was excluded by medical examination and negative intra-operative findings. Written informed consent was obtained from all subjects. The study was approved by the ethical committees of RIKEN and other participating institutions.

**Table 1:** Clinical data of spinal extradural arachnoid cyst subjects.

Subject ID^a^	Age at diagnosis (years)	Sex	Cyst		Surgery	Associated feature^b^
			Number	Location		
P1	64	M	1	T12-L2	−	−
P2	36	M	1	T11-L3	+	−
P3	45	M	1	T12-L2	+	−
P4	60	F	1	T12-L2	+	−
**P5**	50	F	1	T12-L2	+	−
**P6**	38	M	1	T12-L1	+	−
P7	45	F	1	T11-L3	+	−
P8	49	F	1	T11-L2	+	−
**P9**	59	M	1	T11-S	+	−
P10	38	M	1	L2-4	+	−
P11	38	F	1	L2-4	+	−
P12	61	F	2	L2-3/ L5-S	+	−

^a^Subjects harboring *HOXD4* mutations are in bold.

^b^Distichiasis, lymphedema and skeletal anomalies.

### Exome sequencing

We sheared genomic DNA (3 μg) by Covaris S2 system (Covaris) and processed with SureSelect All Exon 5 kit (Agilent Technologies). We sequenced the captured DNAs with HiSeq 2000 (Illumina) with 101 base pair-end reads with seven indices. We performed the image analysis and base calling by HiSeq Control Software/Real Time Analysis and CASAVA1.8.2 (Illumina) and mapped the sequences to human reference genome hg19 by Novoalign 3.00.02 (P1–P7) and Novoalign 3.00.04 (P8–P12). We removed PCR duplication by the Picard tools. The variants were called by Genome Analysis Toolkit (GATK) v1.6–5 or v2.7–4 using the thresholds recommended in GATK best practices v.3 with hand filtering, and annotated by ANNOVAR (2012 February 23).

### Analysis of exome sequence data

We hypothesized that SEDAC was inherited in an autosomal-dominant fashion since all subjects were sporadic. We filtered out these variants with the script created by BITS (Tokyo, Japan) according to following conditions: 1) variants registered in ESP6500, 2) variants found in our in-house controls (n = 575), 3) synonymous changes, 4) non-flagged rare variants registered in dbSNP build 137 (MAF < 0.01), and 5) variants within segmental duplications.

### Sanger sequence

We sequenced PCR products from genomic DNAs and plasmid clones of the expression vectors using 3730xl DNA Analyzer (Applied Biosystems) according to the manufacture’s instruction.

### *in silico* analyses of mutation and gene

To evaluate the variants identified by the sequence analyses, we considered the following variants as deleterious: 1) nonsense variants (stop codon and frame shift), 2) deletion of > 3 amino acid, and 3) missense variants with high *in silico* prediction scores (PolyPhen-2 > 0.95 and SIFT < 0.05). We performed *in silico* structural analysis as previously described.[7, 8] Briefly, since no experimental structure was available for the human HOXD4 homeodomain, we used a crystal structure of the *Antennapedia* homeodomain in complex with DNA (PDB code 9ANT) by searching an analogous structure of human HOXD4 using SWISS-MODEL server. To evaluate the impact of the deletion on the homeodomain structure, we calculated free energy change upon the deletion using the FoldX software (version 3.0β5). We used STRING 9.1. (http://string-db.org/) for *in silico* protein-protein interaction analysis. We examined expression patterns of *Hoxd4* and *Foxc2* in mouse embryo using EMBRYS database (http://embrys.com/).

### Luciferase assay of the recombinant *HOXD4* mutations

We cloned the cDNA of the wild-type *HOXD4* into the *Xba*I and *EcoR*V sites of the pFLAG-CMV-4 expression vector (Sigma Aldrich). We introduced the two *HOXD4* mutations by site-directed mutagenesis using PrimeSTAR Mutagenesis Basal Kit (Takara). For Western blotting, HeLa cells were transfected in a 12-well plate using 500 ng of the HOXD4-pFLAG construct. Transfected HeLa cells were washed with PBS, were harvested by scraping after 48 hours of incubation, and were resolved by SDS-PAGE. The N-terminal FLAG epitope was detected by immunoblot with a mouse anti-FLAG monoclonal antibody (Sigma Aldrich). We cloned the murine *Hoxd4*-responsive luciferase vector into the *Nhe*I and *Bgl*II sites of the pGL3-Basic vector (Promega). The homeodomains of mouse and human HOXD4 have completely identical amino acid sequences.[9] For the luciferase assay, HeLa cells were transfected in a 24-well plate using 150 ng of the HOXD4- pFLAG construct, 300 ng of the luciferase reporter vector and 1 ng of the pRLSV40 control vector (Promega). Transfected cells were grown for 48 hours under 5% CO_2_ at 37°C. The cells were washed with PBS and dissolved in 150 μl of a passive lysis buffer. We performed the dual-luciferase assays using the Promega Dual Luciferase Assay kit (Promega) according to the manufacturer’s protocol.

## Results

### Whole exome sequencing

**Fig 2:**
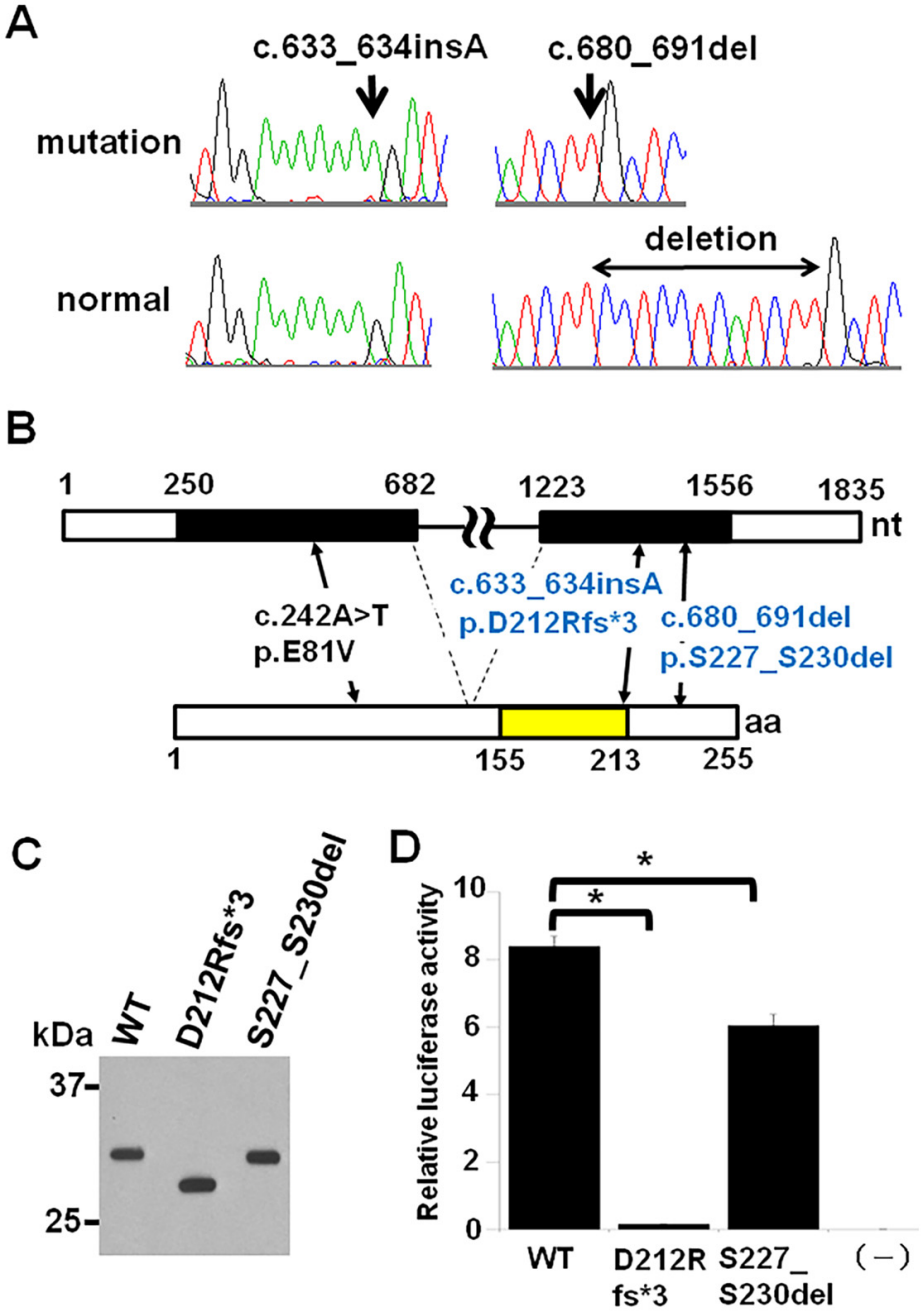
*HOXD4* mutations in spinal extradural arachnoid cyst. (A) DNA sequence. A heterozygous insertion (c.633_634insA) in P5 and a heterozygous deletion (c.680_691) in P6 and P9. Upper and lower sequences represent mutation and normal (reference) sequences, respectively. The double-headed arrow indicates the deletion. (B) Genomic (upper column) and protein (lower column) structures of *HOXD4* and positions of mutations identified in spinal extradural arachnoid cyst (blue letters) and acute lymphoid leukemia (black letters). Exons and homeodomain are black- and yellow-colored, respectively. (C) Western blot analysis. Lysates of transfected HeLa cells were resolved by SDS-PAGE and detected by immunoblot for the N-terminal FLAG epitope. The mutant proteins showed expected size bands. D) Dual luciferase assay. Luciferase activities of WT and mutant vectors. The activities of D212Rfs*3 and S227_S230del HOXD4 were significantly decreased compared to that of WT HOXD4. (–): empty vector, thick bar: mean value, error bar: SD, asterisk: p value < 0.01 (t-test).

The mean depth of coverage for reads was 109.5×, and 92.8% of the targeted bases had sufficient coverage (more than 20 reads on average) (S1 Table). By the WES, we identified heterozygous variants in *HOXD4*, c.633_634insA (p.D212Rfs*3) in P5 and c.680_691del (p.S227_S230del) in P6 and P9. The results were confirmed by the Sanger sequence (Fig 2A). These variants were not found in any public databases including WES database of 1,208 Japanese people (Human Genetic Variation Browser). *HOXD4* is a two-exon gene (Fig 2B) on chromosome 2p. c.633_634insA is considered to lead to a truncation of the HOXD4 protein rather than to cause nonsense-mediated mRNA decay, because the mutation is located in the last exon (Fig 2B). c.680_691del produces a deletion in the C-terminal of the homeodomain (Fig 2B). We then examined the presence of structural abnormalities, such as deletion and duplication, in *HOXD4* using TaqMan real-time quantitative PCR method as previously described;[6] however, no abnormality was found in the subjects (data not shown).

### *in silico* analysis of the *HOXD4* variants

We evaluated the effect of the two *HOXD4* variants on their ability to bind to DNA using *in silico* structural analysis as previously described.[6, 7] c.633_634insA (p.D212Rfs*3) that affects the hydrophobic core of the homeodomain was predicted to disturb the folding of the tertiary structure and thereby impairs DNA binding activity of the homeodomain. No implications could be drawn about the impact of c.680_691del (p.S227_S230del) from the analysis.

### *in vitro* analysis of the transcriptional activities of the *HOXD4* variants

We then analyzed their transcriptional activity of the variants *in vitro*. We confirmed the protein production using a Western blot analysis (Fig 2C) and measured the transactivation activity of the HOXD4 constructs using luciferase assay. The mutant constructs with p.D212Rfs*3 and p.S227_S230del had significantly reduced luciferase activities compared to the wild type (Fig 2D), indicating that the two mutations were loss of function mutations. This result was replicated in HEK293 cells (data not shown).

### *in silico* analysis of the relation of *HOXD4* and *FOXC2*

HOXD4 and FOXC2 are both homeobox transcription factors involved in various developmental processes. To examine the relation of the two transcription factors, we evaluated their interaction using STRING 9.1. We found that HOXD4 and FOXC2 proteins had indirect, but close interactions with each other (S1 Fig). We then investigated the expression patterns of *Hoxd4* and *Foxc2* during early development of the mouse and found that they had similar temporal and spatial expression pattern in the somite of mouse embryo (S2 Fig).

## Discussion

We have found two heterozygous mutations in *HOXD4* in SEDAC. Both are thought to be loss-of-function mutations. To our knowledge, only one *HOXD4* mutation has been reported; a germline mutation, c.242A>T (p.E81V) (Fig 2B) was found in two acute lymphoid leukemia patients, one with skeletal abnormalities (bilateral cervical ribs and L5 sacralization) and another without skeletal abnormalities.[10] The mutation also showed a partial loss of transcriptional activity. The association of SEDAC with acute lymphoid leukemia has not been reported. The phenotype discrepancy between the present and reported mutations may be due to the difference of the position and/or the effect of the mutations. Alternatively, based on the fact that c.242A>T of *HOXD4* in the two leukemia patients is linked to a specific haplotype composed of three variants in other *HOXD* genes (c.746-79_746-68del and c.1025T>G in *HOXD10* and c.557G>A in *HOXD12*), van Scherpenzeel Thim *et al*. speculated that the *HOXD4* mutation is insufficient to confer leukemia susceptibility on its own, necessitating combined action of other *HOXD* variants.[10] In our patients, those HOXD variations and other HOX variants common to the two SEDAC subjects were not found.

Pathomechanism of the SEDAC by *HOXD4* mutations has not been clarified. *HOXD4* belongs to the homeobox (HOX) gene family of transcription factors. *HOX* genes play important roles in morphogenesis and are critical in the establishment of body axes during embryogenesis.[10–12] HOXD4 is involved in determining positional values in the developing spine.[10] The dural defect of SEDAC is considered to be due to abnormal dura mater development. *FOXC2*, the disease gene for syndromic SEDAC[5, 6] also encodes a homeobox transcription factor and expressed in the developing mesodermal mesenchyme which forms the dura mater.[13–15] Dura mater development would be impaired by *FOXC2* loss of function mutations. On the other hand, HOXD4 plays an important role in morphogenesis by determining positional values in the developing spine.[16] In fact, Northern blot analysis revealed that HOXD4 transcripts were expressed in spinal cords of 5–9 week-old human fetuses.[17] HOXD4 may also be involved in dura mater development. Interestingly, *in silico* interaction analysis showed that HOXD4 and FOXC2 proteins had close interactions with each other (S1 Fig). Furthermore, *Hoxd4* and *Foxc2* showed similar spacio-temporal expression patterns in the somite of mouse embryo (S2 Fig). These findings suggest that the regulatory network of the transcription factors containing HOXD4 may be important in dura mater development and involved in the etiology and pathogenesis of SEDAC. The network may include other causal genes of SEDAC.

## Supporting Information

S1 Fig Protein-protein interaction analysis between HOXD4 and FOXC2 protein. Interaction analysis between HOXD4 and FOXC2 proteins using STRING database. Stronger associations are represented by thicker lines. Nodes are colored (if they are directly linked to the input) or white (nodes of a higher iteration) as defined by STRING database. HOXD4 and FOXC2 proteins had indirect, but strong interaction.
(DOCX)

S2 Fig Embryonic gene expression of *Hoxd4* and *Foxc2*. *Hoxd4* (top) and *Foxc2* (bottom) expression in mouse during E9.5–11.5. Pink, dark-yellow, orange, light-yellow, blue, and red represent somite, tail bud, maxillary process, mandibular arch, hyoid arch, and eye, respectively. The stronger color density represents more expression. Both genes had strong and similar expression in the somite.
(DOCX)

S1 Table Summary of the exome sequencing performance. (DOCX)
