# Supplementary material for: Identification of HOXD4 Mutations in Spinal Extradural Arachnoid Cyst
Source: PLoS One. 2015 Nov 6;10(11):e0142126. doi: 10.1371/journal.pone.0142126 (PMC4636324; doi:10.1371/journal.pone.0142126)

**S1 Fig. Protein-protein interaction analysis between HOXD4 and FOXC2 protein.**

Interaction analysis between HOXD4 and FOXC2 proteins using STRING database. Stronger associations are represented by thicker lines. Nodes are colored (if they are directly linked to the input) or white (nodes of a higher iteration) as defined by STRING database. HOXD4 and FOXC2 proteins had indirect, but strong interaction.


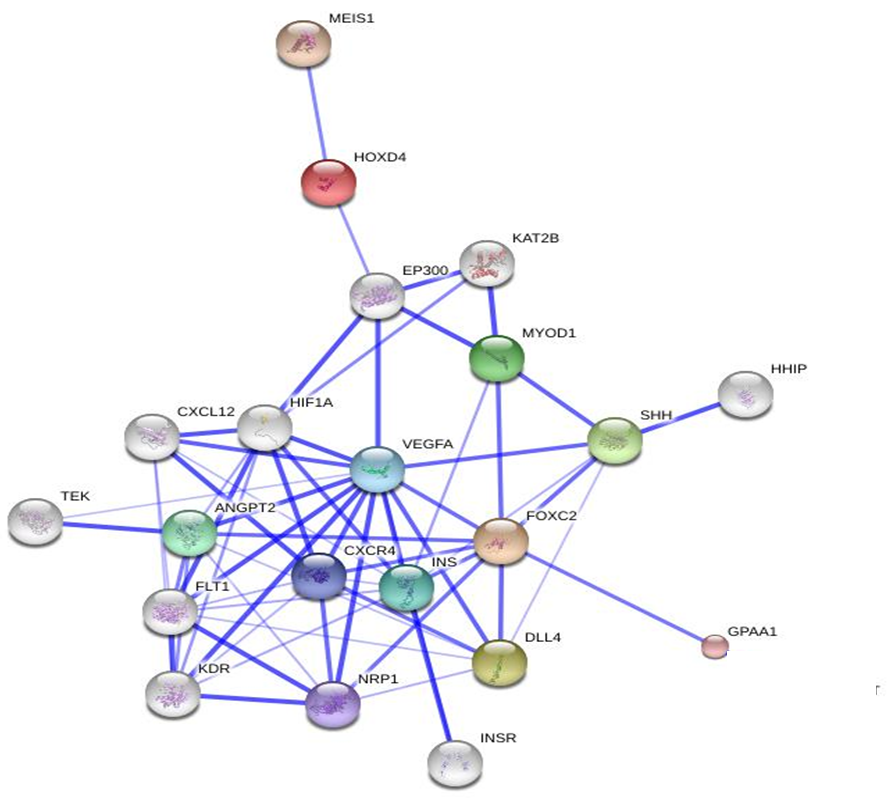

Supplement: S1 Fig — Interaction analysis between HOXD4 and FOXC2 proteins using STRING database. Stronger associations are represented by thicker lines. Nodes are colored (if they are directly linked to the input) or white (nodes of a higher iteration) as defined by STRING database. HOXD4 and FOXC2 proteins had indirect, but strong interaction. (DOCX) [file pone.0142126.s001.docx]
