# Supplementary material for: Identification of HOXD4 Mutations in Spinal Extradural Arachnoid Cyst
Source: PLoS One. 2015 Nov 6;10(11):e0142126. doi: 10.1371/journal.pone.0142126 (PMC4636324; doi:10.1371/journal.pone.0142126)

**S2 Fig. Embryonic gene expression of *Hoxd4* and *Foxc2*.**

*Hoxd4* (top) and *Foxc2* (bottom) expression in mouse during E9.5-11.5.

Pink, dark-yellow, orange, light-yellow, blue, and red represent somite, tail bud, maxillary process, mandibular arch, hyoid arch, and eye, respectively. The stronger color density represents more expression. Both genes had strong and similar expression in the somite.


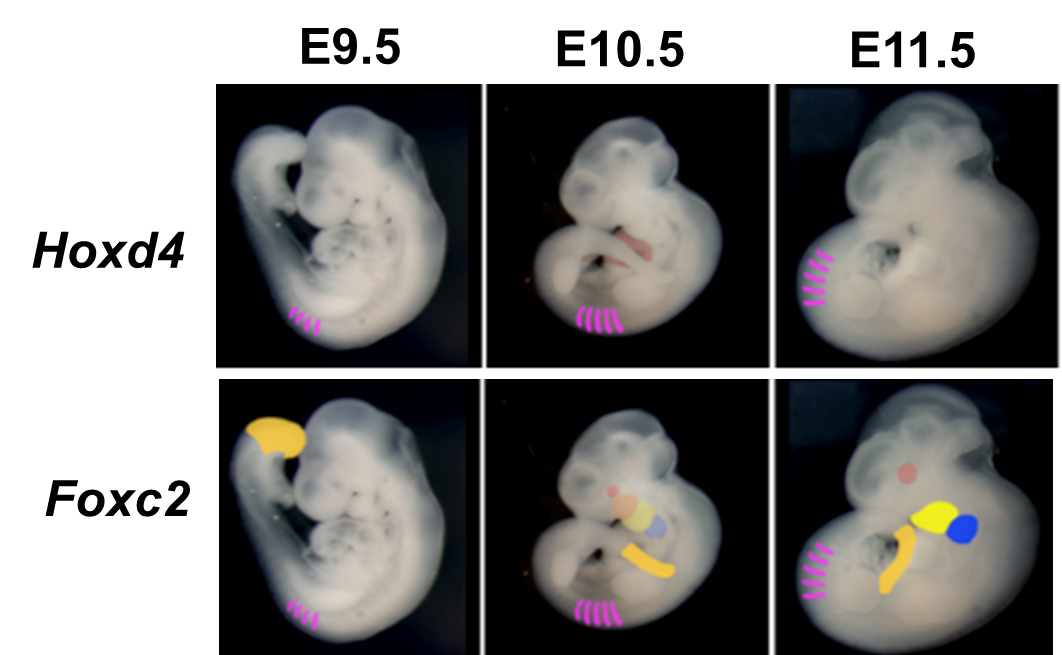

Supplement: S2 Fig — Hoxd4 (top) and Foxc2 (bottom) expression in mouse during E9.5–11.5. Pink, dark-yellow, orange, light-yellow, blue, and red represent somite, tail bud, maxillary process, mandibular arch, hyoid arch, and eye, respectively. The stronger color density represents more expression. Both genes had strong and similar expression in the somite. (DOCX) [file pone.0142126.s002.docx]
