# Supplementary material for: Identification of HOXD4 Mutations in Spinal Extradural Arachnoid Cyst
Source: PLoS One. 2015 Nov 6;10(11):e0142126. doi: 10.1371/journal.pone.0142126 (PMC4636324; doi:10.1371/journal.pone.0142126)
Supplement: S1 Table — (DOCX) [file pone.0142126.s003.docx]

**S1 Table.** Summary of the exome sequencing performance

| **Subject ID** | **No. of bases covered** | **Coverage** | | |
| --- | --- | --- | --- | --- |
|  |  | **average depth** | **above 10x (%)** | **above 20x (%)** |
| P1 | 3,791,558,490 | 113.3 | 96.4 | 94.0 |
| P2 | 4,162,638,768 | 124.4 | 96.4 | 94.2 |
| P3 | 4,230,427,291 | 126.4 | 96.7 | 94.8 |
| P4 | 4,483,875,309 | 134.0 | 95.3 | 92.0 |
| P5 | 3,641,557,771 | 108.8 | 94.8 | 90.6 |
| P6 | 4,247,443,051 | 126.9 | 96.5 | 94.5 |
| P7 | 4,195,642,948 | 125.4 | 96.6 | 94.6 |
| P8 | 3,226,166,717 | 96.4 | 69.2 | 93.3 |
| P9 | 2,824,103,299 | 84.4 | 95.7 | 91.7 |
| P10 | 2,982,739,157 | 89.1 | 95.7 | 91.9 |
| P11 | 3,375,077,011 | 100.8 | 94.9 | 90.4 |
| P12 | 2,832,280,625 | 84.6 | 95.8 | 92.0 |
| Mean | 3,666,125,870 | 109.5 | 93.7 | 92.8 |
